# Supplementary material for: Crop rotation and native microbiome inoculation restore soil capacity to suppress a root disease
Source: Nat Commun. 2023 Dec 8;14:8126. doi: 10.1038/s41467-023-43926-4 (PMC10709580; doi:10.1038/s41467-023-43926-4)
Supplement: Supplementary file 1 — Supplementary Information [file 41467_2023_43926_MOESM1_ESM.pdf]

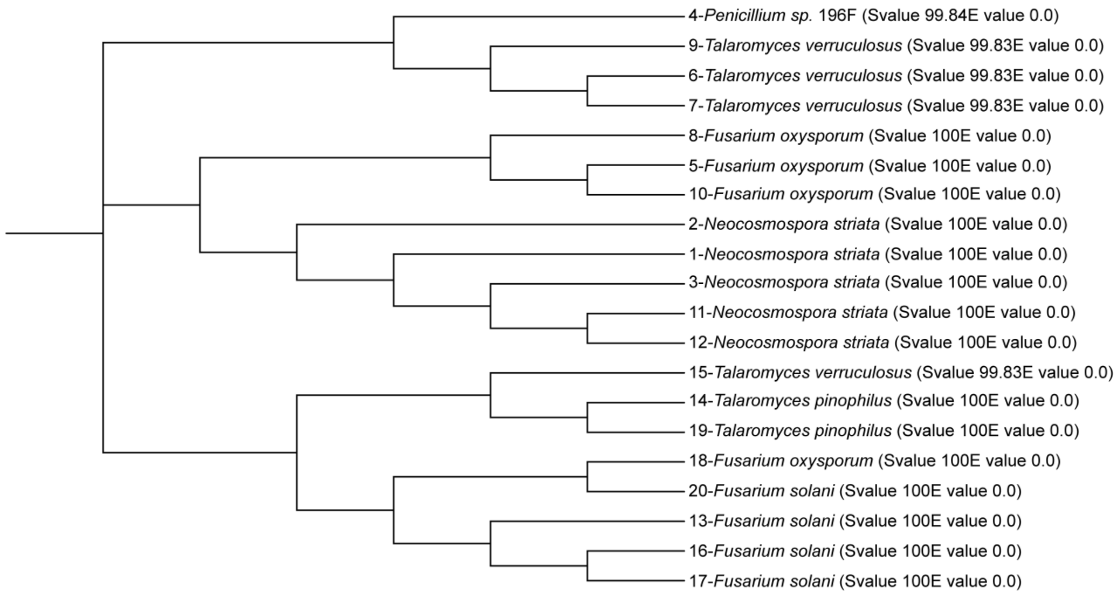

Supplementary Fig. 1 Phylogenetic tree of 20 potential pathogenic fungi isolated and identified.

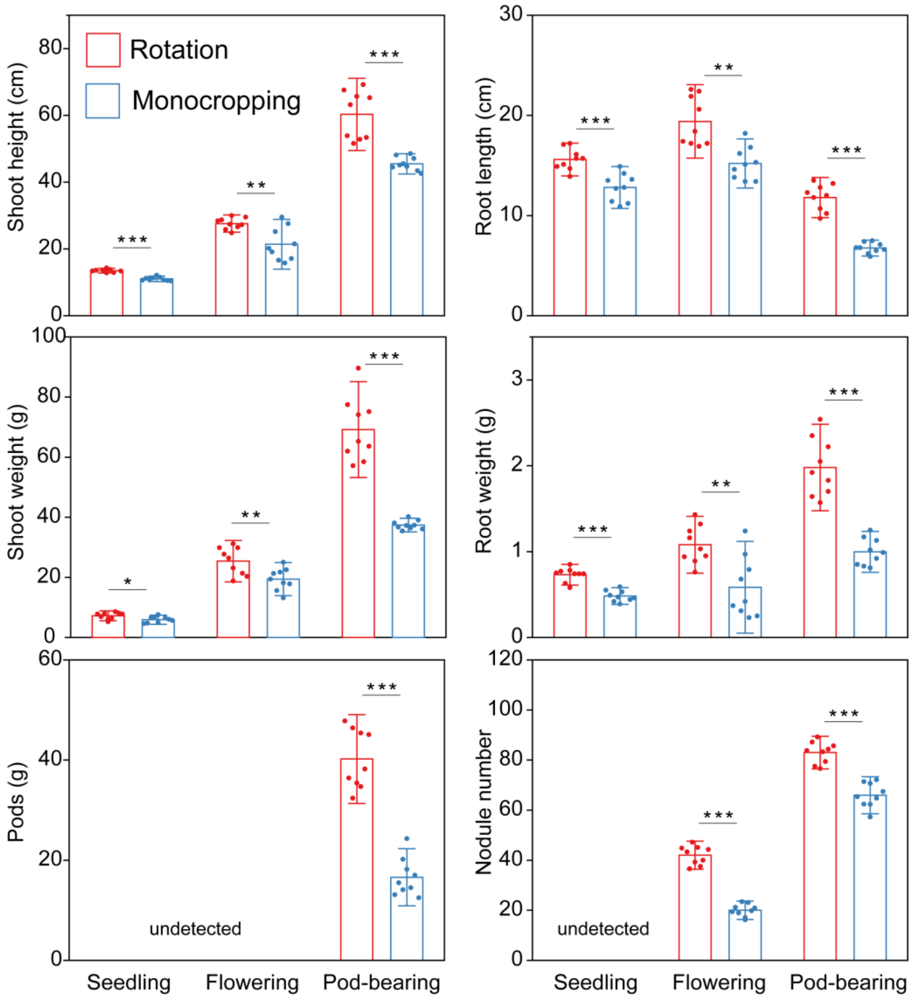

Supplementary Fig. 2 Comparison of the growth index of monocropped and rotation peanut in field experiment. Asterisks above the bars indicate statistically significant differences between treatments based on two-sided tests by Student's t-test (**P* < 0.05, ***P* < 0.01, ****P* < 0.001). Each bars represents the mean ± SD (n=9 biologically independent samples).

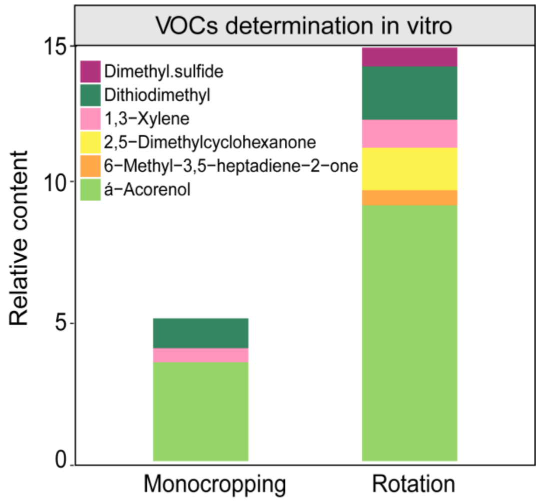

Supplementary Fig. 3 Analysis of potential inhibitory volatile organic compounds (VOCs) produced by cultivable bacteria (two-sided Student's *t* test, $P < 0.05$, n=3 biologically independent samples).

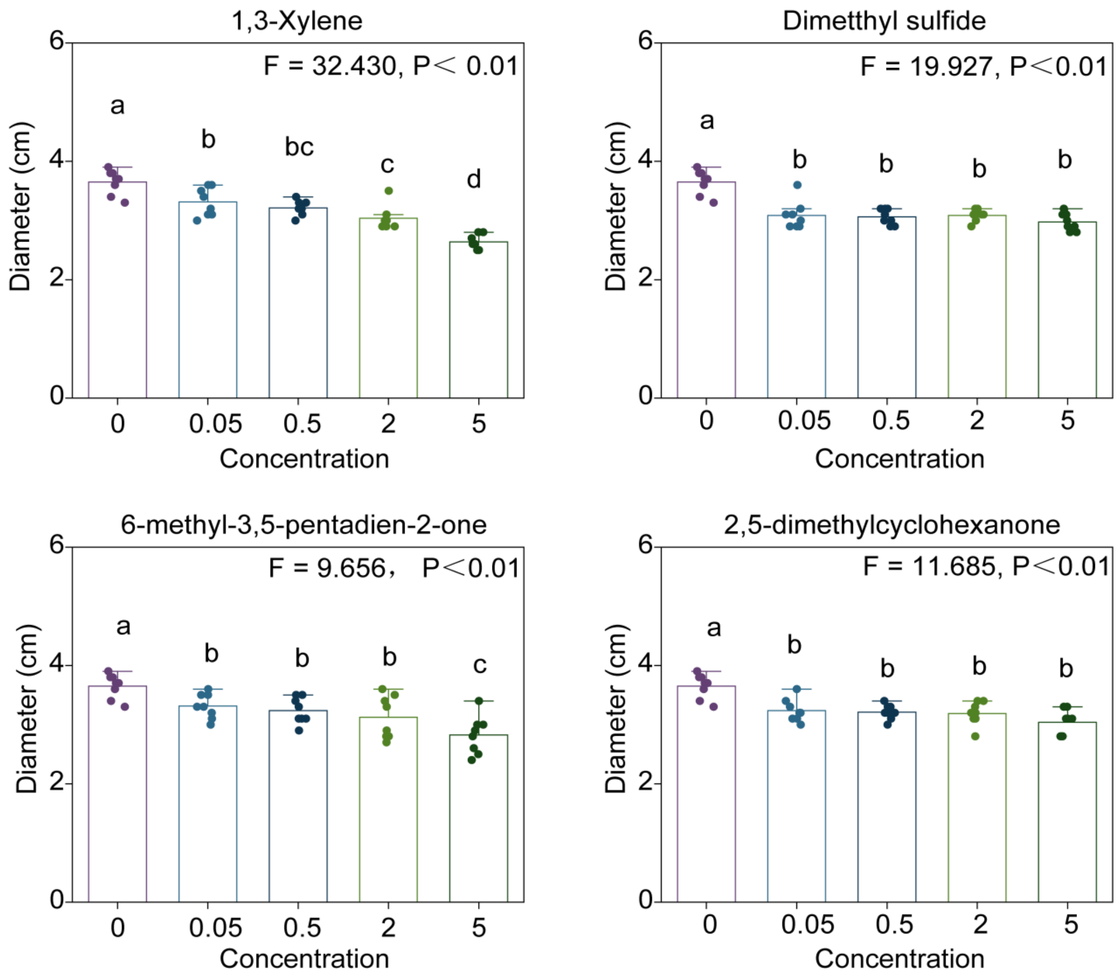

Supplementary Fig. 4 The inhibitory ability of standard VOCs on the development of pathogen at four concentrations. Different numbers above the bars indicate statistically significant differences between the different concentrations according to ANOVA's test based on two-sided (n=8 biologically independent samples). Each bars represents the mean ± SD.

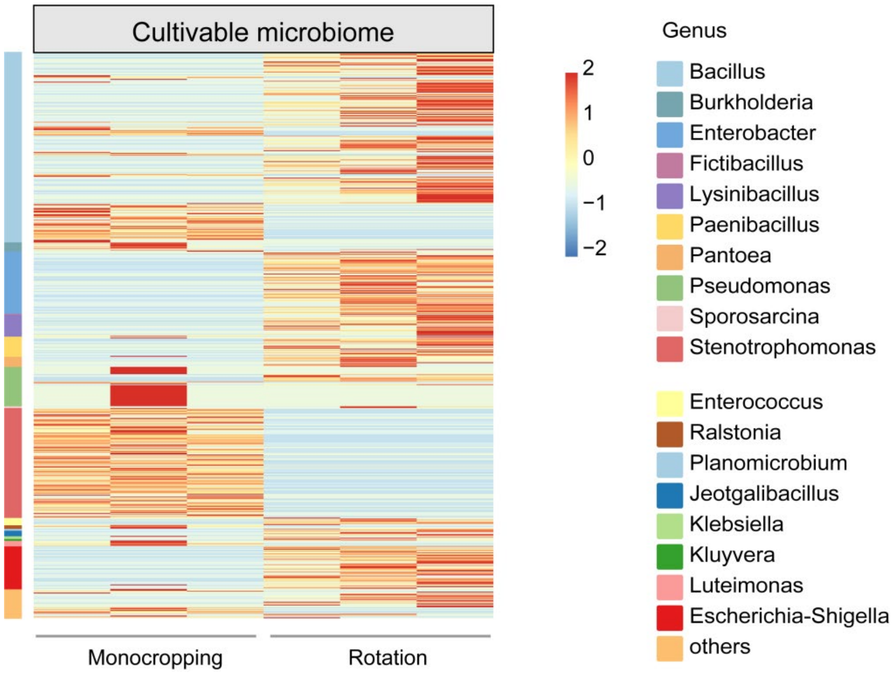

Supplementary Fig. 5 Heatmap of depleted and enriched OTUs in rhizosphere of monocropped peanut ($P < 0.05$, n=3 biologically independent samples).

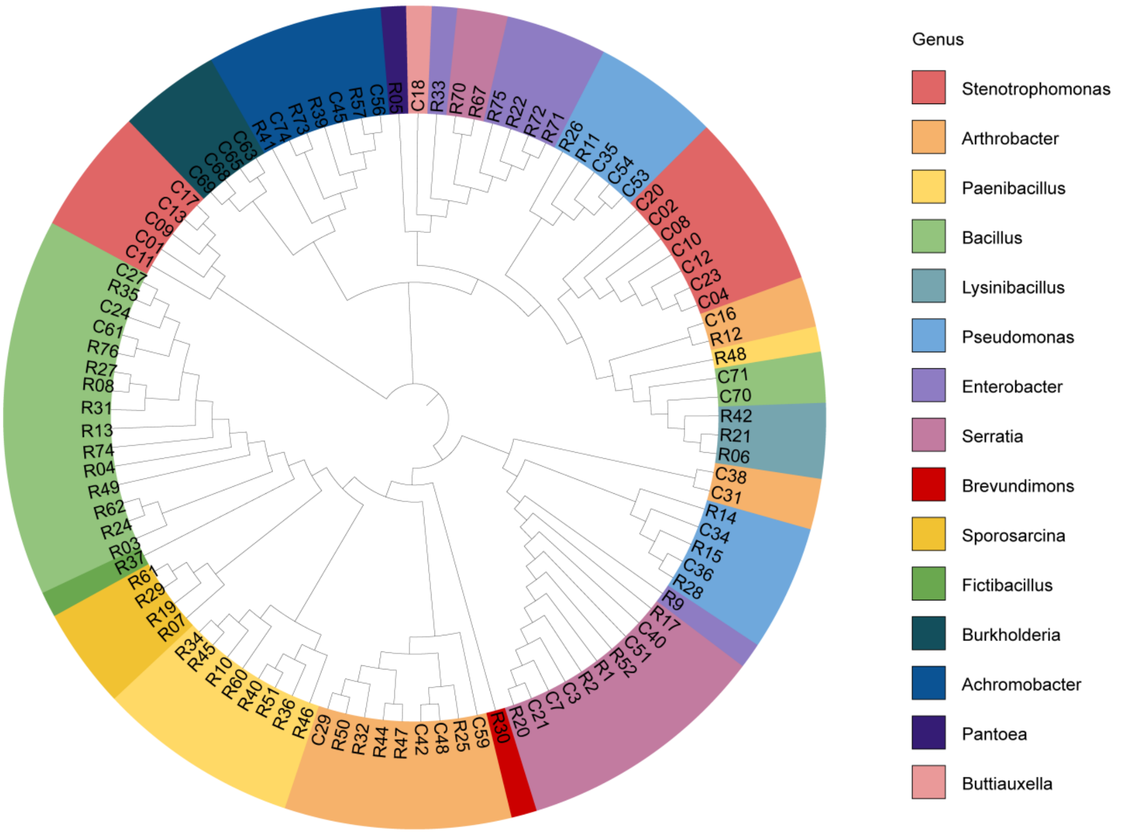

Supplementary Fig. 6 A phylogenetic tree was constructed of 103 strains successfully identified from culturable microbial communities in monocropping and rotation.

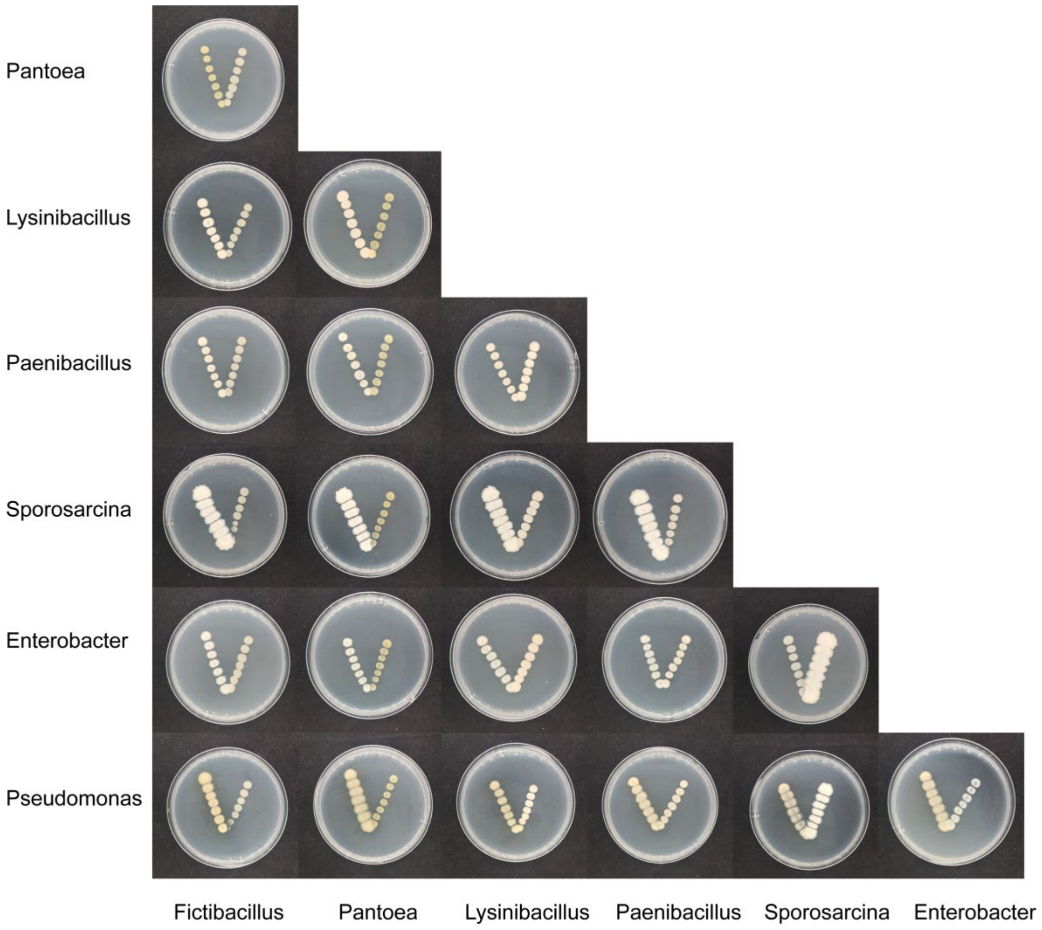

Supplementary Fig.7 Pairwise interactions between depleted strains(n=8 biologically independent samples).

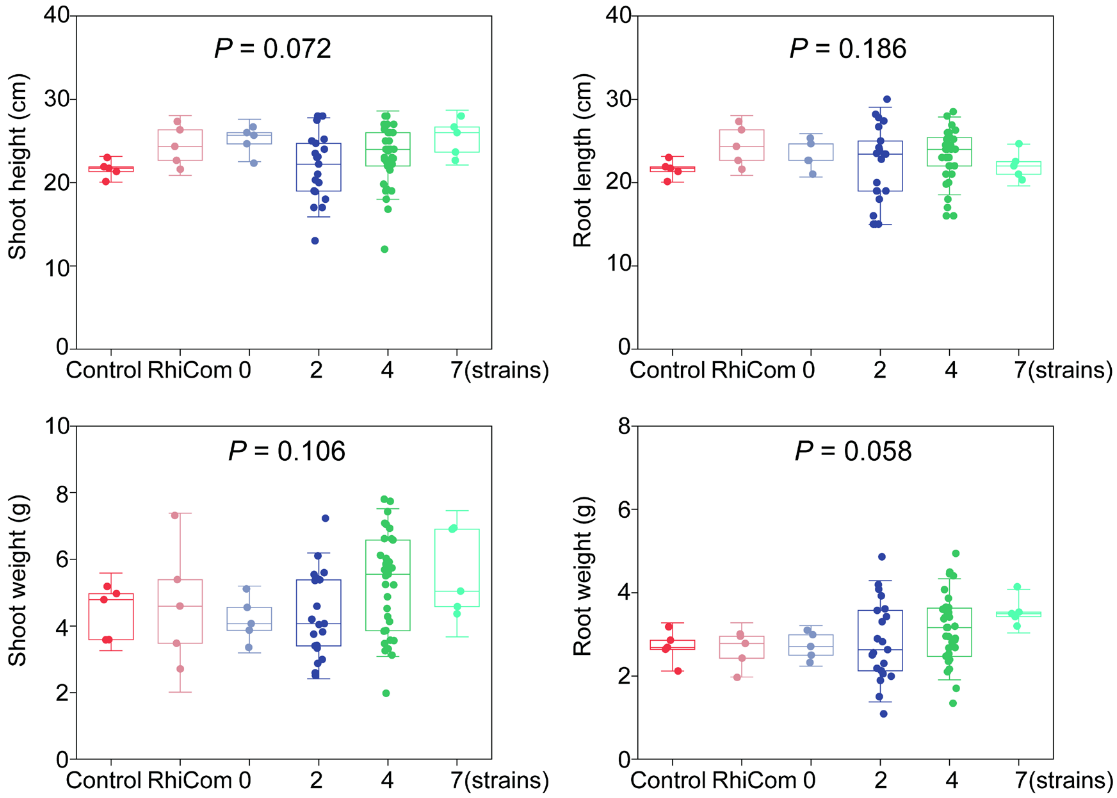

Supplementary Fig. 8 Effects of supplemented monocropping-depleted strains on the growth of peanut seedlings. *P* values were calculated using ANOVA's test based on two-sided ($n_{control}$=5, n_7=5, n_4=35×5, n_2=21×5, n_0=5; biologically independent samples). RhiCom, treatment with monocropping rhizosphere community; SynCom, supplemented synthetic community composed of monocropping-depleted strains to monocropping rhizosphere community; 7, a SynCom composed of 7 depleted strains; 4, SynComs composed of 4 depleted strains; 2, SynComs composed of 2 depleted strains; 0, a SynCom composed of 7 inactivated depleted strains. Each bars represents the mean ± SD. Horizontal bars within boxes represent the median. The tops and bottoms of boxes represent 75th and 25th quartiles, respectively. The upper and lower whiskers represent the range of non-outlier data values.

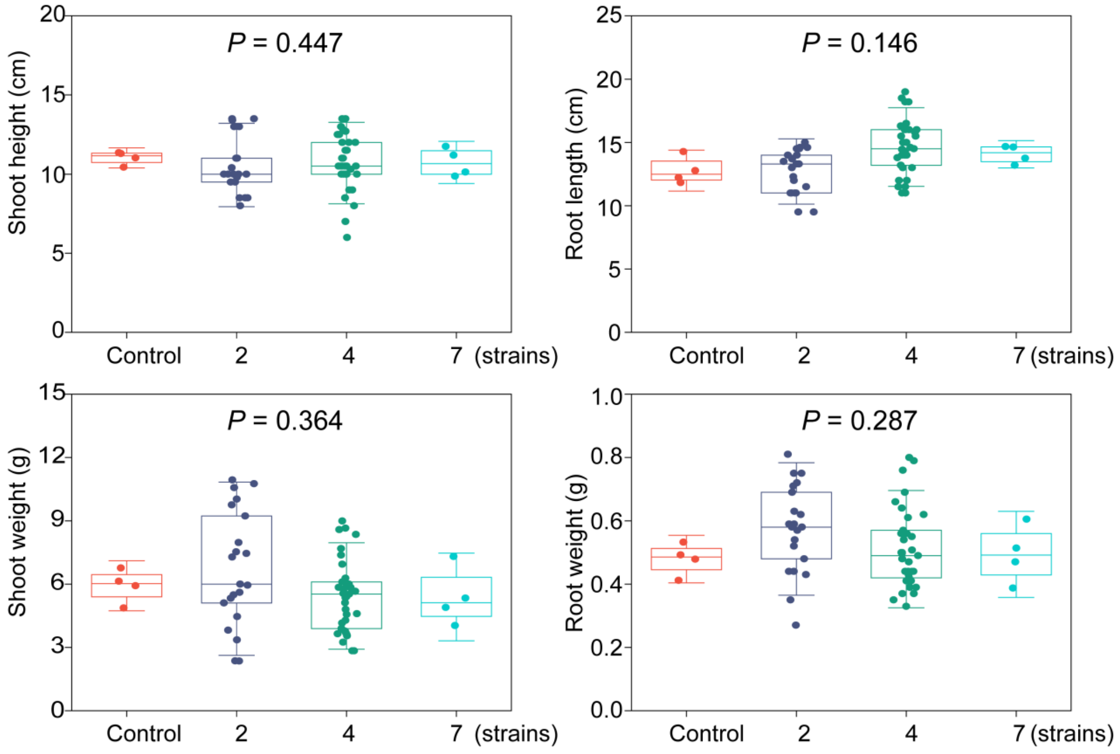

Supplementary Fig. 9 Effect of re-inoculation of different SynComs on peanut growth in a monocropping peanut field. *P* values were calculated using ANOVA's test based on two-sided ($n_{control}$=4, n_7=4, n_4=35×4, n_2=21×4; biologically independent samples). Control: without inoculating the SynComs. 7, a SynCom composed of 7 depleted strains; 4, SynComs composed of 4 depleted strains; 2, SynComs composed of 2 depleted strains. Each bars represents the mean ± SD. Horizontal bars within boxes represent the median. The tops and bottoms of boxes represent 75th and 25th quartiles, respectively. The upper and lower whiskers represent the range of non-outlier data values.

Supplementary Table 1 Analysis of pathogen suppression and growth-promoting characteristics of depleted bacteria and enriched bacterial stains in monocropping peanut rhizosphere.

Strain names	Siderophore	Organic Phosphorus	Inorganic Phosphorus	IAA(mg/L)	Inhibitory
Pantoea sp.	++	—	—	18.9±2.31	—
Fictibacillus sp.	—	+	—	8.4±1.54	+
Enterobacter sp.	+++	++	+	35.7±5.02	+
Paenibacillus sp.	—	—	—	28.4±2.36	+++
Sporosarcina sp.	—	+	—	5.36±0.54	+++
Lysinibacillus sp.	—	—	—	25.2±5.63	—
Pseudomonas sp.	—	—	—	7.83±0.75	+
Stenotrophomonas sp.	—	—	—	15.2±2.31	+
Burkholderia sp.	++	+	+	10.0±1.54	—

Inhibitory: The direct inhibitory effect of bacteria on the hyphae of *F. oxysporum*; + means that there are characteristics, and the more plus signs, the stronger the characteristics; - means that there are no characteristics.

Supplementary Table 2 The combination modes of 7, 4 and 2 depleted bacteria to construct synthetic community.

Strains number	Combination modes		
7	ABCDEFG		
4 (35 combinations)	ABCD	ABCE	ABCF
	ABCG	ABDE	ABDF
	ABDG	ABEF	ABEG
	ABFG	ACDE	ACDF
	ACDG	ACEF	ACEG
	ACFG	ADEF	ADEG
	ADFG	AEFG	BCDE
	BCDF	BCDG	BCEF
	BCEG	BCFG	BDEF
	BDEG	BDFG	BEFG
	CDEF	CDEG	CDFG
	CEFG	DEFG	
2 (21 combinations)	AB	AC	AD
	AE	AF	AG
	BC	BD	BE
	BF	BG	CD
	CE	CF	CG
	DE	DF	DG
	EF	EG	FG

A: *Pantoea* sp.; B: *Fictibacillus* sp.; C: *Enterobacter* sp.; D: *Paenibacillus* sp.; E: *Sporosarcina* sp.; F: *Lysinibacillus* sp.; G: *Pseudomonas* sp..
