# Supplementary material for: Crop rotation and native microbiome inoculation restore soil capacity to suppress a root disease
Source: Nat Commun. 2023 Dec 8;14:8126. doi: 10.1038/s41467-023-43926-4 (PMC10709580; doi:10.1038/s41467-023-43926-4)
Supplement: Supplementary file 3 — Reporting Summary [file 41467_2023_43926_MOESM3_ESM.pdf]

nature portfolio

Corresponding author(s): Xiaogang Li

Last updated by author(s): Nov 10, 2023

Reporting Summary

Nature Portfolio wishes to improve the reproducibility of the work that we publish. This form provides structure for consistency and transparency in reporting. For further information on Nature Portfolio policies, see our Editorial Policies and the Editorial Policy Checklist.

Statistics

For all statistical analyses, confirm that the following items are present in the figure legend, table legend, main text, or Methods section.

n/a	Confirmed	
☐	☒	The exact sample size (*n*) for each experimental group/condition, given as a discrete number and unit of measurement
☐	☒	A statement on whether measurements were taken from distinct samples or whether the same sample was measured repeatedly
☐	☒	The statistical test(s) used AND whether they are one- or two-sided<br>*Only common tests should be described solely by name; describe more complex techniques in the Methods section.*
☐	☒	A description of all covariates tested
☐	☒	A description of any assumptions or corrections, such as tests of normality and adjustment for multiple comparisons
☐	☒	A full description of the statistical parameters including central tendency (e.g. means) or other basic estimates (e.g. regression coefficient) AND variation (e.g. standard deviation) or associated estimates of uncertainty (e.g. confidence intervals)
☐	☒	For null hypothesis testing, the test statistic (e.g. *F*, *t*, *r*) with confidence intervals, effect sizes, degrees of freedom and *P* value noted<br>*Give P values as exact values whenever suitable.*
☒	☐	For Bayesian analysis, information on the choice of priors and Markov chain Monte Carlo settings
☒	☐	For hierarchical and complex designs, identification of the appropriate level for tests and full reporting of outcomes
☒	☐	Estimates of effect sizes (e.g. Cohen's *d*, Pearson's *r*), indicating how they were calculated

Our web collection on statistics for biologists contains articles on many of the points above.

Software and code

Policy information about availability of computer code

Data collection	No software was used for data collection.
Data analysis	All the softwares used for analysis have been described in the Online Methods. All software used in this study included: R v4.1.0, MEGA v5, Cutadapt v1.2.1, Trinity v2.2.0, Bowtie2 v2.2.9, RSEM v1.3.0, MEGAN 5, https://www.omicstudio.cn/tool/11, https://itol.embl.de/, https://doi.org/10.6084/m9.figshare.24558613.

For manuscripts utilizing custom algorithms or software that are central to the research but not yet described in published literature, software must be made available to editors and reviewers. We strongly encourage code deposition in a community repository (e.g. GitHub). See the Nature Portfolio guidelines for submitting code & software for further information.

Data

Policy information about availability of data

All manuscripts must include a data availability statement. This statement should provide the following information, where applicable:

- Accession codes, unique identifiers, or web links for publicly available datasets
- A description of any restrictions on data availability
- For clinical datasets or third party data, please ensure that the statement adheres to our policy

The raw reads from Illumina sequencing and metatranscriptome generated in this study have been deposited in the NCBI database under accession no. PRJNA1029910(https://www.ncbi.nlm.nih.gov/search/all/?term=PRJNA1029910) and PRJNA1029900(https://www.ncbi.nlm.nih.gov/search/all/?

term=PRJNA1029900), respectively. Additional data generated in this study are provided in the Supplementary Information/Source Data file. The data used for this study are available in 'figshare' with the identifier https://doi.org/10.6084/m9.figshare.24558613. For databases used in this study, SILVA database is available at https://www.arb-silva.de; UNITE database is available at https://unite.ut.ee/repository.php.

Research involving human participants, their data, or biological material

Policy information about studies with human participants or human data. See also policy information about sex, gender (identity/presentation), and sexual orientation and race, ethnicity and racism.

Reporting on sex and gender	This information has not been collected.
Reporting on race, ethnicity, or other socially relevant groupings	This information has not been collected.
Population characteristics	This information has not been collected.
Recruitment	This information has not been collected.
Ethics oversight	This information has not been collected.

Note that full information on the approval of the study protocol must also be provided in the manuscript.

Field-specific reporting

Please select the one below that is the best fit for your research. If you are not sure, read the appropriate sections before making your selection.

☐ Life sciences ☐ Behavioural & social sciences ☒ Ecological, evolutionary & environmental sciences

For a reference copy of the document with all sections, see nature.com/documents/nr-reporting-summary-flat.pdf

Ecological, evolutionary & environmental sciences study design

All studies must disclose on these points even when the disclosure is negative.

Study description	We use field, greenhouse and laboratory experiments to investigate the effect of management (monocropping and rotation) on the capacity of rhizosphere microbiomes in suppressing peanut root rot disease. We found that rhizosphere microbiomes in monocultures are less able to suppress fungal pathogens compared to crop rotations, and that inoculating certain microbes can mitigate it.
Research sample	For field experiment, three plots (replicates) of the two cropping regimes were laid out in a randomized block design. From 2012, experimental plots were managed under two cropping regimes (treatments): peanut monocropping and rotation. For monocropping, peanuts were continuously planted from 2012-2018. For rotation, peanut was grown first (2012), and then maize (Zea mays L.), potato (Solanum tuberosum), and soybean (Glycine max) were ordinally planted in every other peanut planting year. The disease index of peanut was investigated at the flowering stage in 2012, 2014 and 2016. At 2018, peanut disease was investigated at seedling, flowering and pod-bearing stage. In the 2018 growing season, plants were removed from the plots and soil samples were collected to determined bacterial communities. For pot experiment, six independent replicates from monocropping and rotation regimes were used for subsequent isolation of the rhizosphere bacterial colonies, the assessment of fungal pathogen suppression, and metatranscriptome analysis. To determine the ability of monocropping depleted strains to resist pathogen invasion, we constructed synthetic communities and performed antagonistic, pot, and field experiments, based on 4-8 replicates.
Sampling strategy	For field experiments, 540 plants (6 plots, 3 time points) were removed from the plots for evaluation of the severity of root rots in the 2018 growing season. At seedling, flowering and pod-bearing stage, 10 peanuts were randomly selected from each plot, and rhizosphere soil of every 5 peanut plants was fully mixed into a single composite soil sample. In total, 36 soil samples (6 plots, 3 time points) were collected. For pot experiment, the rhizosphere soil from 10 pots per plot was pooled, with a total of 6 replicates for both treatments. For others experiments, we set up 3-12 replicates for each experiment to ensure the accuracy of the data.
Data collection	The root rot symptoms were assessed by using a scale of 0-4. Soil bacterial and fungal communities were sequenced using the Illumina MiSeq platform (Illumina, USA) at Majorbio Bio-Pharm Technology Co. Ltd. (Shanghai, China). Quantitative real-time PCR (qPCR) was used for the quantification of fungal abundance. The suppression of a fungal pathogen by the rhizosphere soil was determined by VOC-mediated and directed microcosm antagonism assays. Cultivable rhizosphere microbiome function was analyzed through Metatranscriptome sequencing on a Illumina HiSeq 4000 platform (Shanghai, China). Soil microbial volatile compounds were analyzed using GC(GC-CP3800 and CP-8, Agilent Technologies, USA). The ability of key bacteria to produce IAA was evaluated using Salkowski colorimetric agent. The Chrome Azurol-S (CAS) agar plate method was used to determine the siderophore production capacity of key strains. Pikovskaya (PVK) agar plates was used to determine the siderophore production capacity of phosphate solubilization. Bacterial interactions were performed to determine if the depleted strains were mutually reinforcing or antagonistic. To investigate whether the monocropping-depleted strains have protection effect on plant disease defenses, antagonism, pot and field experiments were performed. All data were recorded by Yanyan Zhou and Zhen Yang.
Timing and spatial scale	The evaluation of the severity of root rot and the sampling of rhizosphere soil were carried out in Yingtan, Jiangxi Province, China. We

Timing and spatial scale	assessed the effect of monocropping on the severity of root rot in June 2012,2014 and 2016 (flowering period). Next, the impact of growth period on root rot severity was assessed in May, June and July 2018. The rhizosphere soil samples were collected every other month from May to July 2018, and rhizosphere soil samples were obtained at seedling stage, flowering stage and pod stage. Experiments of laboratory and microcosm were performed from Sep 2018 to May 2019 in Nanjing, Jiangsu Province, China.
Data exclusions	In bioinformatic analysis of root endosphere fungi and rhizosphere bacteria, to be conservative, low-quality sequences (<150 bp long, with an average quality score <25) were removed. In metatranscriptome sequencing of culturable rhizosphere microbiome, five samples out of six passed quality control after sequencing; sample “C1” yielded very low sequence counts and was not included in the analysis.
Reproducibility	For Illumina sequencing, DNA PCR amplification was triplicated for each sample. For other experiments, we set up 3-10 technical replicates for each treatment to ensure the accuracy of the data.
Randomization	Samples were randomly collected from field station without a priori expectations that the sampling would influence the analysis. In addition, SynCom application in field experiment adopts random block design.
Blinding	No blinding was applied as no statistical tests were performed where blinding could be applied.

Did the study involve field work? ☒ Yes ☐ No

Field work, collection and transport

Field conditions	A field experiment was conducted at the field station of the Chinese Academy of Sciences (Jiangxi Province, China). The soil at the study site is classified as Udic Ferrosol (FAO classification). The field experiment included two cropping regimes (treatments): (1) monocropping with peanut; and (2) rotation, with a 2-year rotation of peanut alternated with other crops.
Location	Field experiments were performed at a field station of the Chinese Academy of Sciences, Jiangxi Province, China (28°130′ N, 116°550′ E).
Access & import/export	The samples were collected from our established experimental plots, with explicit permission from local and national authorities.
Disturbance	No disturbance was caused by the study.

Reporting for specific materials, systems and methods

We require information from authors about some types of materials, experimental systems and methods used in many studies. Here, indicate whether each material, system or method listed is relevant to your study. If you are not sure if a list item applies to your research, read the appropriate section before selecting a response.

Materials & experimental systems

n/a	Involved in the study	
☒	☐	Antibodies
☒	☐	Eukaryotic cell lines
☒	☐	Palaeontology and archaeology
☒	☐	Animals and other organisms
☒	☐	Clinical data
☒	☐	Dual use research of concern
☐	☒	Plants

Methods

n/a	Involved in the study	
☒	☐	ChIP-seq
☒	☐	Flow cytometry
☒	☐	MRI-based neuroimaging

Dual use research of concern

Policy information about dual use research of concern

Hazards

Could the accidental, deliberate or reckless misuse of agents or technologies generated in the work, or the application of information presented in the manuscript, pose a threat to:

No | Yes

- [x] No [ ] Yes — Public health
- [x] No [ ] Yes — National security
- [x] No [ ] Yes — Crops and/or livestock
- [x] No [ ] Yes — Ecosystems
- [x] No [ ] Yes — Any other significant area

Experiments of concern

Does the work involve any of these experiments of concern:

No | Yes

- [x] No [ ] Yes — Demonstrate how to render a vaccine ineffective
- [x] No [ ] Yes — Confer resistance to therapeutically useful antibiotics or antiviral agents
- [x] No [ ] Yes — Enhance the virulence of a pathogen or render a nonpathogen virulent
- [x] No [ ] Yes — Increase transmissibility of a pathogen
- [x] No [ ] Yes — Alter the host range of a pathogen
- [x] No [ ] Yes — Enable evasion of diagnostic/detection modalities
- [x] No [ ] Yes — Enable the weaponization of a biological agent or toxin
- [x] No [ ] Yes — Any other potentially harmful combination of experiments and agents

Plants

Seed stocks: The peanut seeds were harvested from the field trial plots and were harvested at the peanut maturity stage in 2018.

Novel plant genotypes: No new genotypes were involved.

Authentication: No new genotypes were involved.
